# Education and Symptom Reporting in an mHealth App for Patients With Cancer: Mixed Methods Development and Validation Study

**DOI:** 10.2196/60169

**Published:** 2025-04-28

**Authors:** Carolina Muñoz Olivar, Miguel Pineiro, Juan Sebastián Gómez Quintero, Carlos Javier Avendaño-Vásquez, Pablo Ormeño-Arriagada, Silvia Palma Rivadeneira, Carla Taramasco Toro

**Affiliations:** 1 PhD in Health Sciences, Faculty of Medicine, Antonio Nariño University, Bogotá, 111511, Colombia; 2 Center for Cancer Prevention and Control (CECAN), Santiago, Chile; 3 Faculty of Engineering, Institute of Technologies for Innovation in Health and Well-being, Andrés Bello University, Viña del Mar, Chile; 4 Energy Transformation Center, Faculty of Engineering, Andrés Bello University, Santiago, Chile; 5 Faculty of Nursing, Antonio Nariño University, Bogotá, Colombia; 6 School of Nursing, Industrial University of Santander, Bucaramanga, Colombia; 7 Faculty of Engineering, Business, and Agro-Environmental Sciences, Department of Computer Civil Engineering, Universidad Viña del Mar, Viña del Mar, Chile; 8 Facultad de Medicina, Escuela de Enfermería, Pontificia Universidad Católica de Chile, Santiago, Chile; 9 UC-CHRISTUS Health Network, Santiago, Chile

**Keywords:** cancer, patient-reported outcome measures, software design, unpleasant symptom, toxicity, mHealth, mobile health, surveys and questionnaires, application, design, evaluation, chemotherapy, health care communication, mixed methods, validation, efficiency, security

## Abstract

**Background:**

The widespread prevalence of cancer across the globe demands cutting-edge solutions for its treatment. Current cancer therapies, notably chemotherapy, pose challenges due to their side effects. The early detection and management of the side effects are vital but complex. This study introduces a mobile health app designed to bridge the communication gaps between patients with cancer and health care providers. Hence, it allows patients to report symptoms immediately and also enables proactive symptom management by health care providers.

**Objective:**

This study has 2 objectives: first, to design a cancer-focused mobile health app that integrates educational content and real-time symptom reporting for chemotherapy patients. Second, to validate and evaluate the app quality using the Mobile App Rating Scale (MARS). The app seeks to foster health care communication, reduce hospital readmissions, and optimize symptom management, contributing to a more impactful patient experience.

**Methods:**

This mixed-methods study details the development and validation of mobile health applications. The app was designed by a multidisciplinary team, including nurses, medical professionals, pharmaceutical chemists, computer engineers, and software developers, using agile methodologies. For validation, the app was assessed by 13 evaluators, including clinical professionals (nurses and physicians) and engineers. The evaluation included technical performance analysis using Google tools and quality assessment using the MARS, which measures engagement, functionality, aesthetics, and information quality.

**Results:**

Performance metrics highlighted areas for improvement, with loading times showing delays in displaying content. Meanwhile, the response time of the app was moderate, and visual stability remained excellent. The app achieved an overall MARS score of 3.75 (SD 0.42), indicating consistent quality, with functionality scoring the highest (4.35; SD 0.52) and engagement the lowest (3.31; SD 0.61). The reliability of the MARS was confirmed (interclass correlation coefficient: 0.84; 95% CI: 0.72‐0.92). Evaluators unanimously praised the app’s potential benefits for patients and clinical professionals while identifying areas for improvement such as customization, onboarding guidance, and navigation.

**Conclusions:**

The CONTIGO app showed strengths in functionality, usability, and information quality, supported by robust security measures. However, areas such as user interactivity and engagement require improvement. Future refinements will integrate insights from patients with cancer to address user-specific needs and enhance the oncology care experience.

## Introduction

According to the World Health Organization (WHO), cancer is one of the leading causes of mortality worldwide. In 2020, approximately 19 million people were diagnosed, and 9.3 million people die each year [1,2]. In the particular context of Chile, a comparable pattern was noted; in the same year, 54,000 cases of cancer were diagnosed, with 28,000 fatalities attributed to the disease annually. Additionally, cancer is the leading cause of disease burden and is responsible for 13% of disability-adjusted life years (DALYs) [3-6].

Continuing medical advances and the current therapeutic approach to cancer are multimodal and incorporate various strategies to maximize treatment efficacy. This comprehensive approach encompasses modalities such as radiotherapy, surgery, immunotherapy, and chemotherapy, the latter considered the gold standard [7-11]. Additionally, the application of an interdisciplinary approach includes palliative care to reduce suffering and improve the overall quality of life of patients and their families. This multidimensional strategy seeks to address the disease and provide comprehensive support, considering the patient’s physical, spiritual, and psychosocial dimensions throughout the disease [12-14].

In this sense, it is essential for health care professionals to monitor and accompany patients and continues to be a challenge for health care professionals, requiring collaborative efforts between clinical staff, patients, and their families. The WHO promotes ensuring quality in care processes and incorporating patient-reported outcome measures (PROMs) and patient-reported experience measures (PREMs) [15] with tools that allow for the timely identification of these symptoms to contribute positively to patient well-being and service delivery by clinical staff [16].

Significant growth in health care technologies may favor this process. According to projections by the Center for Technology and Communication, approximately 7.49 billion people worldwide will be using mobile devices by 2025 [17] and Android users currently have access to 3.55 million apps [18], of which 1 million are aimed at influencing people’s health, fitness, nutrition, and well-being [19].

Mobile applications in health, known as mHealth apps, have proven helpful and significant benefits for patients and health care professionals. These apps assist patients in adopting healthier lifestyles, enhancing self-care, and improving the quality of services, with a substantial focus on educational services and sensibilization programs [20-22]. This information is crucial for promptly notifying health professionals so that they can make informed decisions and effectively manage patients’ adverse effects [23-27].

In line with these advancements, Wasserman et al [28] conducted a study to evaluate the quality and usefulness of 17 digital mHealth applications designed for adults with cancer. The study focused on assessing apps with tailored digital features and measured their performance using standardized tools, including the Mobile App Rating Scale (MARS) and a checklist of user-desired features derived from the literature. These features included functionalities such as symptom tracking, treatment management, and communication tools, highlighting the potential role of mHealth apps in supporting comprehensive cancer care and addressing unmet needs in symptom management and patient engagement.

We developed the CONTIGO app, designed specifically for the Chilean context and to operate within the UC-Christus network [29]. Its primary aim is to help patients adopt healthier lifestyles, improve their self-care, and positively impact their quality of life and that of their families. In addition, the app aims to facilitate communication between health care professionals and patients to reduce hospital readmissions and unnecessary emergency room visits while optimizing symptom management.

This study presents the results of designing a mHealth application for patients with cancer that integrates educational content with the functionality of immediate notification of unpleasant symptoms associated with chemotherapy administration to the treating physician and nurse. Additionally, it shows the results of validation and quality assessment of the mobile application “CONTIGO” by using the MARS, which allows mHealth developers to maximize the application’s benefits to significantly impact the patient [22,30-36].

## Methods

### Study Type

This study employed a mixed methods approach. This methodology is presented in two sections: (1) design a mobile application, and (2) validation and quality assessment.

### Ethical Considerations

The research protocol for designing a health mobile application for patients with cancer was reviewed and approved by the Scientific Ethics Committee CEC Med-UC (09/2023) ID 230808005. All participants provided informed consent before submitting their responses. Participation was voluntary, and participants were informed of their right to withdraw at any time without consequence. The study did not include any vulnerable populations and was conducted in accordance with established ethical standards for human subjects research. Data were collected anonymously through a Google Form, and no personally identifiable information was recorded. As a result, all data are fully anonymized and cannot be linked to individual participants. Researchers did not have access to responses while participants completed the evaluation, ensuring both privacy and impartiality. No financial or material compensation was offered for participation. Anonymized data may be made available upon request

### mHealth App Design

To develop “CONTIGO,” a multidisciplinary team consisting of nurses, pharmaceutical chemists, medics, computer engineers, and developers was formed. Previous research was considered, and principles of agile methodologies were adopted, such as software development, responsiveness to change, an iterative process with the client, and the assessment of individuals and interactions in the process. As a result, 5 stages for the development of the application were established.

#### Phase 1: Information Analysis

To construct the educational content, we conducted focus groups involving 1 PhD student in health science, a nurse with a master’s degree, 3 nurses with oncology experience, and 2 oncologists. These professionals identified a critical gap in patient education: newly diagnosed oncology patients often forget or lack access to the educational information provided during consultations. This application was developed as a response to this need, ensuring that patients have a reliable and accessible source of information to revisit at their convenience. The research team adhered to the recommendations outlined in the Guide of Elaboration and Application of Focus Groups (G-DEP-005) [37] and followed an iterative process to obtain a theoretical framework for each section of the application. The framework was presented using text, images, and personalized videos. The application’s initial focus prioritized the needs and preferences of health care professionals because the tool was designed to support their educational role with patients. The requirements were collected through a detailed request form completed by a trained clinical team member, who classified them by priority. This approach ensured the application’s alignment with clinical standards while laying the groundwork for future integration of patient-specific preferences.

#### Phase 2: Design

We considered a previously created interface [38], which had been evaluated in earlier studies using patient focus groups. Feedback from these studies informed the decision to adopt and optimize this interface for the current application. Adjustments were made to the graphical interface, focusing on scenario optimization and comprehensive software restructuring. These modifications aimed to ensure an attractive, user-friendly, and efficient interface for the application’s target users.

Architectural development prioritized asynchronous services to enhance system flexibility and scalability, ensuring the application could adapt to future updates and user demands.

#### Phase 3: Development

In this phase, decisions were made regarding implementation and coding, scenario adjustment, and software restructuring. Docker containers were used to simplify deployment and optimize application management and distribution. Security measures, such as management through Cloudflare, were integrated to prevent potential distributed denial-of-service (DDoS) attacks. Data encryption techniques were also applied to ensure information security. An open-source, high-performance PostgreSQL relational database management system was defined. A backup program was established every 3 hours using incremental backup copies to ensure data integrity and availability.

#### Phase 4: Functional Testing

This phase focused on verifying the system’s robustness through emulation and simulation tests, followed by the implementation of the application on real devices. The teams were divided into 2 groups: one consisting of engineers and developers and the other consisting of a clinical team. This division aimed to enhance mobile device applications’ quality, stability, and user experience.

#### Phase 5: Deployment

**Figure 1.**
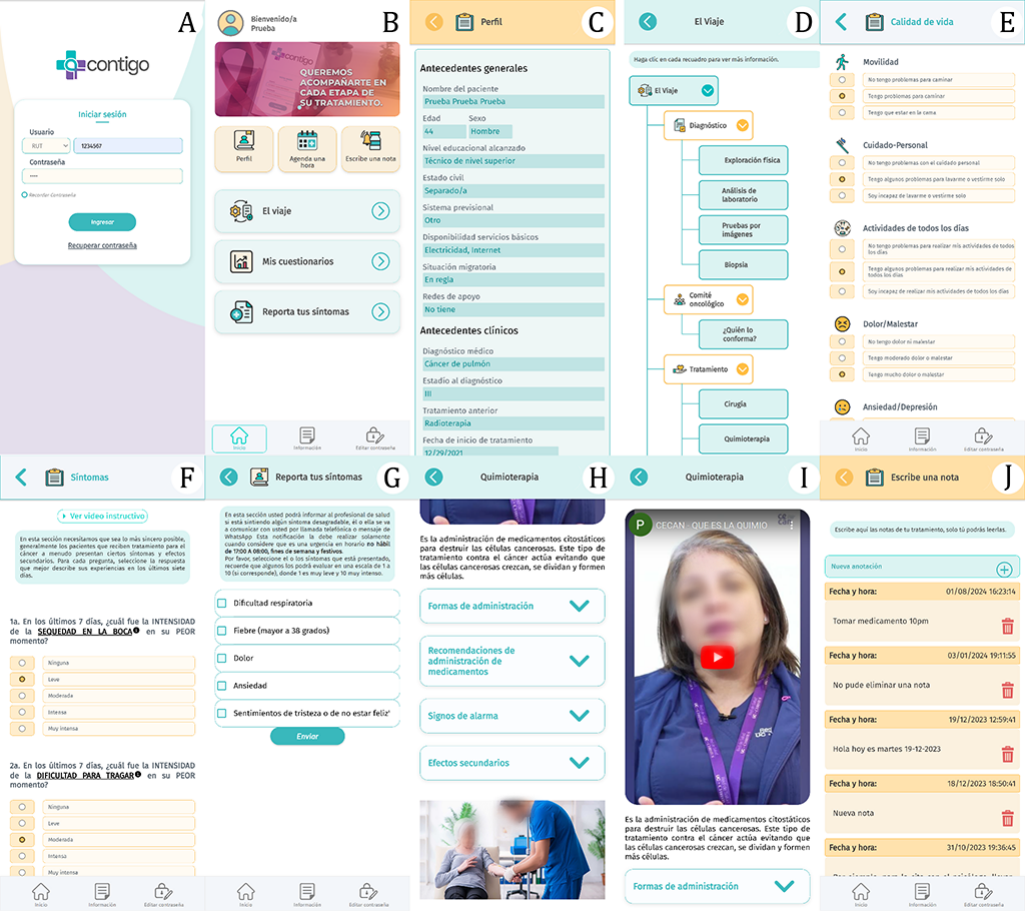
“CONTIGO” mHealth app Interface: (**A**) login screen, (**B**) home screen, (**C**) patient profile, (**D**) the journey section, (**E**) quality of life questionnaire, (**F**) PRO-CTCAE toxicity detection questionnaire, (**G**) symptom reporting module, (**H**) educational content (text and images), (**I**) educational content (videos), and (**J**) notes section.

The engineering team officially delivered the “CONTIGO” mobile application to proceed with the final validation process and quality evaluation. Figure 1 presents the final version of the application and its sections, which include a variety of features designed to support patients and clinical professionals.

The application allows patients to record chemotherapy-related symptoms, enabling clinical teams to make data-driven decisions and manage side effects more effectively. Furthermore, the app offers features such as patient profile management, appointment scheduling, and access to educational materials presented through text, images, and videos.

The educational videos included in the app cover a variety of topics designed to improve patients’ understanding of their treatment and care. These topics include laboratory tests, imaging tests, oncology committee decisions, treatment options, chemotherapy, radiotherapy, immunotherapy, palliative care, what is cancer, what is chemotherapy, what is a catheter with a reservoir, what is a continuous infusion pump, what are side effects, and what are the warning signs. The videos aim to provide patients with clear and concise explanations of medical procedures and key concepts to empower them in managing their care.

Additionally, the app includes digital questionnaires for reporting physical and psychological symptoms, as well as assessing quality of life. To enhance patient-clinician communication, the app integrates an instant messaging system, facilitating real-time interaction with the clinical team.

### Validation and Quality Assessment

In this study, the validation strategy reported in TGUM-GISSIC groups was applied and adapted to the current Chilean context in Chile. Technical attributes that determine the quality of an application, such as performance, graphical interface structure, usability, robustness, and information security, were identified [39]. Recommendations followed from the assessment and accreditation of health-related mobile applications [20]. The literature search began by identifying a validated questionnaire, and among the identified instruments, the MARS scale was selected.

The performance and robustness of the application were evaluated using Lighthouse [40,41], a widely used, open-source Google-developed tool for assessing web and mobile app quality. Lighthouse provides metrics across several categories, such as performance, accessibility, best practices, and search engine optimization (SEO), which are essential for understanding the application’s functionality and user experience.

Lighthouse simulates a page load in a controlled environment, emulating device and network conditions to capture performance data and generate metrics for performance. These insights highlight areas needing improvement, ultimately helping developers enhance the user experience.

Its metrics include:

- First contentful paint (FCP): Measures the time it takes for the first visible content, such as text or images, to appear on the screen after the page starts loading. It provides an early indication to users that the page is loading and responsive, enhancing perceived performance. Optimal values are under 1.8 seconds, moderate values range between 1.8 and 3.0 seconds, and values above 3.0 seconds indicate a need for improvement.
- Largest contentful paint (LCP): Represents the time taken for the largest visible content, like a banner image or block of text, to fully render in the viewport. It reflects how quickly the main content becomes available, which is critical for user engagement. Optimal values are under 2.5 seconds, moderate values range between 2.5 and 4.0 seconds, and values above 4.0 seconds require improvement.
- Speed index: It evaluates how quickly content is visually displayed during the loading process by calculating the average time for visible portions of the page to appear. This metric gives a holistic view of the loading experience, beyond individual milestones. Optimal values are under 3.4 seconds, moderate values range between 3.4 and 5.8 seconds, and values above 5.8 seconds indicate poor performance.
- Total blocking time (TBT): Measures the total duration when long tasks (exceeding 50 ms) block the main thread, preventing the app or website from responding to user inputs. This metric directly impacts the perceived interactivity of the app during its loading phase. A lower TBT score indicates smoother transitions and better responsiveness. TBT scores were categorized based on established benchmarks: scores under 200 milliseconds were considered optimal, scores between 200 and 600 milliseconds as moderate, and scores above 600 milliseconds as requiring improvement.
- Cumulative layout shift (CLS): Assesses the visual stability of a page by measuring unexpected layout shifts caused by late-loading elements like images or ads. A low CLS score ensures a smooth and stable user experience, avoiding accidental clicks or frustrating movements of content. Optimal CLS values are under 0.1, while scores between 0.1 and 0.25 indicate moderate stability. Values above 0.25 reflect significant layout instability, which can negatively impact user experience.

The MARS scale was adapted and validated in Spanish to evaluate other attributes of “CONTIGO” critically and systematically [30-32]. This scale consists of 23 items, offering a reliable, flexible, multidimensional, Likert-type tool, with scores ranging from 1 (worst) to 5 (best). It comprises 3 sections. The first one evaluates unbiased quality, consisting of 4 dimensions (engagement, functionality, aesthetics, and quality of information). The second one assesses subjective quality, and the last one evaluates with 6 questions designed the perceived impact of the application on the users and their behavior change.

### Application Evaluation

For the evaluation process of the “CONTIGO” mobile application, eligibility criteria were established for potential evaluators. This aims to ensure that evaluators possess the necessary expertise to provide meaningful feedback on the application’s functionalities and effectiveness in supporting patients. The evaluators were selected from convenience samples that included nurses with master’s degrees or specialization in oncology, computer engineers with prior experience in mobile application development, and professionals working in various oncology settings who have at least 3 years of practical experience. The sample included professionals from institutions directly involved in the app’s development as well as external institutions. This approach ensured a diversity of perspectives during the evaluation process.

Evaluators who were directly involved in the development of the “CONTIGO” mobile application were excluded.

The unit of analysis for this research comprises the responses collected through the application of the MARS, provided by clinical oncology experts and computer engineers who evaluated the quality of the mobile application designed for patients with cancer.

The guidelines from the CHERRIE Checklist were followed for the survey application in Google Form [42].

### Procedure for MARS Questionnaire Evaluation

Professionals meeting the criteria were invited to participate in the evaluation. During the evaluation period, which spanned approximately 1 month, each evaluator had independent access to the application to explore its features and functionalities at their convenience.

To ensure consistent understanding of the evaluation criteria, each evaluator received an initial training session with a researcher. During this session, researchers explained the elements of the MARS tool using official explanatory slides from the tool’s authors. After this, evaluators independently used the app and completed the MARS rating questionnaire anonymously through a Google Form in Spanish. Informed consent was obtained before submission.

To further support the evaluators, researchers were available to answer questions or clarify doubts during the evaluation period. However, researchers could not access the responses while evaluators completed the questionnaire, ensuring the anonymity and impartiality of the process.

### Data Analysis

The free-text comments provided by evaluators were analyzed using thematic analysis, following Braun and Clarke’s [43] approach. This method allowed us to systematically identify recurring patterns and key themes from the qualitative feedback.

To enhance the reliability of the analysis, 2 independent researchers reviewed the comments and performed an open coding process. The initial codes were then discussed and refined through a consensus-based approach to consolidate the final themes. The analysis focused on 3 main areas: design and usability, content and features, and user onboarding.

No qualitative analysis software was used; instead, a manual coding process was conducted to ensure a direct focus on the key user experience concerns highlighted by the evaluators.

## Results

### App Development

During the early stages of app development, focus groups with health care professionals identified a key challenge: newly diagnosed oncology patients often forget or lack access to the educational information provided during consultations. This finding reinforced the need for a digital tool that allows patients to revisit educational materials at their convenience. As a result, the app was designed to integrate structured educational content through text, images, and videos, ensuring accessibility and continuous support for patients.

### Performance and Robustness

**Figure 2.**
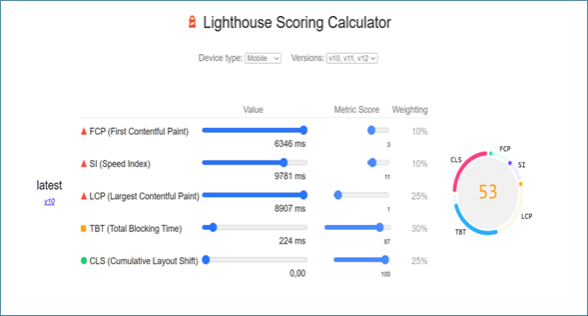
Performance (Source: Chrome Web Store. Lighthouse).

The performance evaluation highlights areas requiring improvement, with an FCP of 6.3 seconds and an LCP of 8.9 seconds, indicating significant delays in rendering the initial and largest visible content. The Speed Index (SI) of 9.8 seconds reflects slow visual load times, impacting the perceived user experience. Despite these results, the TBT of 224 milliseconds falls within the moderate range, suggesting acceptable interactivity during loading. Visual stability, measured by CLS, scored 0, ensuring a smooth and stable interface (Figure 2).

**Figure 3.**
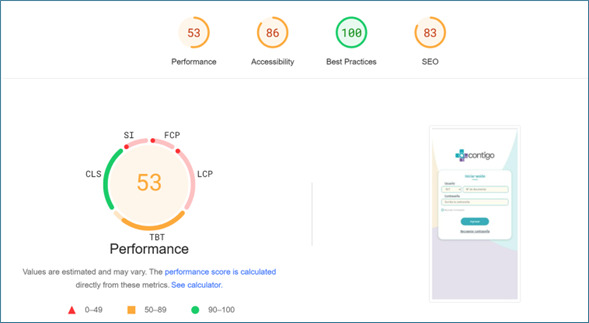
Performance and robustness CONTIGO mobile application.

In addition to these detailed performance metrics, the app was evaluated more broadly across categories such as Accessibility, Best Practices, and SEO (Figure 3). Accessibility scored 86, reflecting good compliance with accessibility standards. Best Practices achieved a perfect score of 100, indicating full adherence to technical and security guidelines, while SEO scored 83, highlighting solid SEO with some room for improvement. Figure 3 provides a general overview of the app’s technical quality, complementing the specific performance insights presented in Figure 2.

It is important to note that the data presented in Figures2  3 were obtained using automated testing tools, specifically the Lighthouse tool (Chrome Web Store). These evaluations were conducted to simulate realistic usage conditions and assess the app’s technical performance, including speed, visual stability, and compliance with industry standards.

### Security

The security certificate was issued by a trusted entity, “R3.” This certificate indicates the validity of both server and client authentication in secure communication. A series of hexadecimal characters evidence the certificate’s integrity, its fingerprint. With this certificate, the server identity can be identified when the user uses the mobile application, thereby thwarting phishing attempts and guaranteeing those users connect to the correct server.

### Mobile App Rating Scale (MARS)

The MARS, which consists of 3 sections and 4 dimensions, was used to evaluate other technical aspects of quality, including content, usability, functionality, operability, accessibility, and reliability.

In total, 13 experts assessed the CONTIGO app: 6 were clinical staff, including nurses and doctors, and 7 were informatics engineers. In terms of educational level, 23.1% held a PhD, 15.4% were specialists, 30.85% had a master’s degree, and 30.8% only had an undergraduate degree. The mean age of the experts was 41 years, providing a balanced representation of professionals across different stages of their careers.

The overall quality of the app, evaluated as the mean MARS score, was 3.75 out of 5 (SD 0.42) (Table 1). Next, we proceeded to assess each of the 4 dimensions of the MARS. The mean score for objective quality was 3.82 (SD 0.42), whereas subjective quality was lower at 3.38 (SD 0.72), with a higher variation as expected. The objective quality comprises 4 dimensions. Functionality had the highest score at 4.35 (SD 0.52), followed by information at 3.87 (SD 0.45), aesthetics at 3.85 (SD 0.59), and engagement with an average score of 3.31 (SD 0.61). Lastly, the app’s impact on habits of life received a score of 3.96 (SD 0.55).

**Figure 4.**
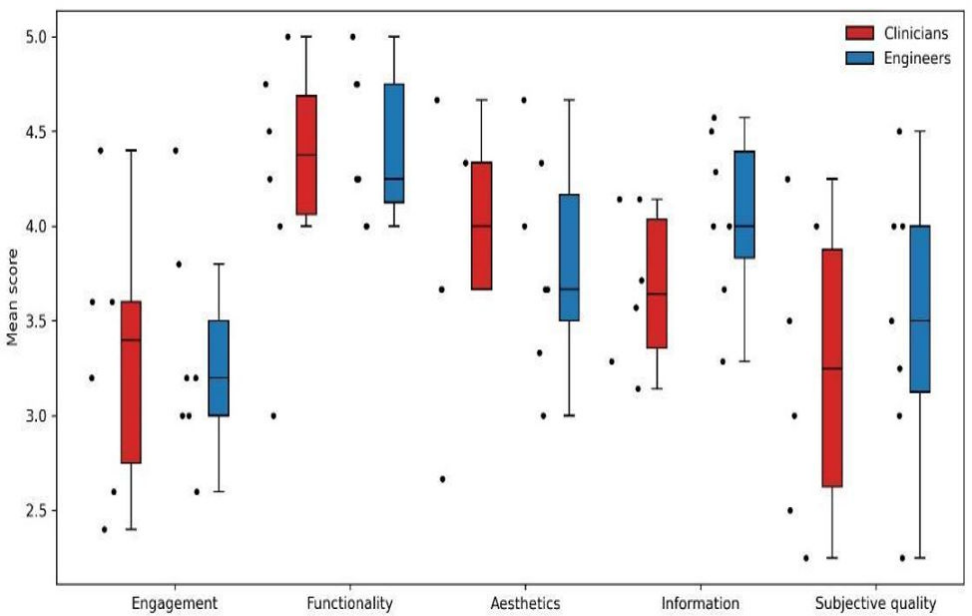
Boxplot comparing clinicians versus engineers.

To see if engineers and clinicians had different opinions about the app, we analyzed the statistics for each group individually. We did not find significant differences in the scores given by these 2 groups of evaluators (Figure 4).

**Table 1.** Mobile application rating scale of CONTIGO.

	Mean	SD	IQR
Objective quality	3.82	0.42	0.48
Engagement	3.31	0.61	0.60
Entertainment	3.08	0.83	1.00
Interest	3.46	1.08	1.00
Customization	2.85	1.23	1.00
Interactivity	3.23	0.70	0.00
Target group	3.92	0.47	0.00
Functionality	4.35	0.52	0.75
Performance	4.69	0.61	0.00
Ease of use	4.23	0.58	1.00
Navigation	4.08	0.92	1.00
Gestural design	4.38	0.62	1.00
Aesthetics	3.85	0.59	0.67
Layout	3.77	0.89	1.00
Graphics	3.77	0.70	1.00
Visual appeal: How good does the app look?	4.00	0.68	0.00
Information	3.87	0.45	0.57
Accuracy of the app description	3.38	1.27	1.00
Goals	3.36	1.23	1.50
Quality of information	4.08	0.47	0.00
Quantity of information	4.00	0.55	0.00
Visual information	4.08	0.73	1.00
Credibility	4.08	0.83	2.00
Evidence base	4.00	0.00	0.00
Subjective quality	3.38	0.72	1.00
Would you recommend this app?	3.85	1.17	2.00
How many times do you think you would use this app?	3.54	0.75	1.00
Would you pay for this app?	2.38	1.21	2.00
What is your overall star rating of the app?	3.77	0.80	1.00
Habits of life	3.96	0.55	0.33
Awareness	4.15	0.77	1.00
Knowledge	4.38	0.74	1.00
Attitudes	3.69	0.72	1.00
Intention to change	3.92	0.83	0.00
Seek of help	3.77	0.70	0.00
Behavior change	3.85	0.53	0.00

Consequently, with the above, the MARS demonstrated good reliability among evaluators with an interclass correlation coefficient (0.84; 95% CI 0.72‐0.92)). The total score demonstrated high internal consistency (Cronbach α=.86). Although the MARS has been previously validated, reporting these metrics ensures that the evaluators in this study demonstrated consistent scoring. The Pearson correlation revealed a moderate but significant correlation between the MARS star rating item (#23), r(13)=0.657, *P*<.01.

Upon completing each questionnaire, experts were allowed to voluntarily provide comments about the app. Unanimously, they agreed that the application’s design would be highly beneficial for both patients and clinical professionals, particularly in aiding in the early detection of symptoms.

The free-text comments were formally recorded and analyzed using thematic analysis to identify key areas for improvement. A total of 12 comments were collected, and 3 main themes emerged:

- Design and usability: Suggestions included enhancing font readability, improving navigation, and ensuring consistency in icon usage across different sections. For instance, one evaluator noted: "The font size is too small in some sections, making it difficult to read. Consistent font sizes for text would improve usability.”
- Content and features: Evaluators recommended adding more detailed symptom tracking options and tailoring educational content to different user needs. A clinical evaluator suggested: “The app could include fields for tracking specific chemotherapy cycles and whether the treatment is curative or palliative.”
- User onboarding: Some participants highlighted the need for better guidance for first-time users, such as an introductory tutorial. One comment stated: “Adding a quick tutorial for new users would help them navigate the app more effectively.”

## Discussion

### Principal Findings and Comparison With Previous Works

This article describes the development and initial evaluation of a mobile application designed to support oncology patients undergoing chemotherapy and assesses its quality. The findings emphasize the importance of interdisciplinary collaboration and iterative design processes in health care app development. These processes ensure that the app’s content is robust, its usability is optimized, and it aligns with clinical and educational standards [37].

Therefore, the results provide a detailed insight into the performance and overall quality of the application. While the app demonstrated strengths in areas such as accessibility (86) and adherence to best practices (100), it also highlighted areas for improvement in performance metrics, including FCP (6.3 s) and LCP (8.9 s), which exceeded optimal thresholds. The TBT scored within the moderate range (224 ms), reflecting acceptable interactivity, and the CLS scored 0, ensuring excellent visual stability. Overall, the application adheres to crucial quality standards [20,39,40] but underscores the need for further optimizations to enhance perceived user performance and loading times. These findings emphasize the importance of iterative testing and refinement during app development.

One important usage of MARS is to evaluate which areas of the app require more work to provide the final user with a better experience. In that line, we reviewed the score of each item and looked for the reasons behind that score, taking into account comments given about the app.

Of the app’s 4 subjective dimensions, engagement received the lowest overall score, indicating significant room for improvement. This was largely due to limited customizability (2.85), as the app lacks options for important capabilities such as color and font size customization, which are particularly useful for users with reduced vision or color blindness, as well as direct access to functionality through favorite shortcuts.

Additionally, the entertainment subdimension scored 3.08, reflecting the app’s reliance on informative videos as its main source of entertainment and the absence of gamification elements or other engaging digital features. However, the interest subdimension scored slightly higher at 3.46, suggesting that the app maintained a moderate level of engagement among evaluators despite these shortcomings. Concerning the functionality, the app showed the highest score. Evaluators appreciated its good performance (4.69), gestural design (4.38), ease of use (4.23), and ease of navigation (4.08). The aesthetics of the app (3.85) were evaluated moderately high; some users noticed that there were some inconsistencies in the layout, graphics, and fonts.

The app performed well in the Information dimension (3.87), especially in items such as the quality of information (4.08), visual information (4.08), credibility (4.08), quantity of information (4.00), and evidence base (4.00). Its performance in terms of the accuracy of the app description (3.38) and its goals (3.36), on the other hand, was much lower. The app was not available on the app store; the description provided in the Google form questionnaire given to the evaluators was taken into account for the assessment. While some users were highly positive, stating they would recommend the app to everyone and rating it as one of the best they have used, others were more reserved or critical.

The subjective quality of the application varied among users based on their recommendations. Even though the app received a good score in terms of how likely evaluators would recommend the app (3.38), at the same time it received a much lower score in terms of how likely evaluators would pay for the app (2.38).

The results also showed that the evaluators consider that app would likely have a good impact on the habits of life (3.96). The evaluators provide a critical foundation for understanding the app’s strengths and areas for improvement [20,30,31]. This study highlights the value of involving health care professionals in the early stages of app evaluation, as their expertise ensures alignment with clinical standards and educational goals. Additionally, it is crucial to involve patients to capture the unique needs and preferences of the app’s end-users.

To address this limitation, 2 ongoing clinical trials will directly involve patients with cancer [44,45]. These trials will evaluate the app using the uMARS tool, which focuses on patient-centered aspects such as usability, engagement, and impact on quality of life. By integrating patient feedback, these trials aim to refine the app further, ensuring it meets the specific needs of its target population.

Despite this limitation, the study contributes to the field of health apps by demonstrating the importance of phased evaluation approaches. These ensure that health apps are rigorously tested for technical and clinical standards before involving end-users, creating a strong foundation for iterative improvement.

### Conclusions

This study provides an initial evaluation of the CONTIGO app, a tool designed to support oncology patients undergoing chemotherapy by addressing key aspects of symptom tracking, education, and patient-clinician communication.

The findings demonstrate that the app meets critical standards for functionality, usability, and information quality, supported by robust security measures and exceptional visual stability. However, performance evaluations revealed areas requiring improvement, particularly in loading times and user interactivity, emphasizing the need for further technical optimizations to enhance the user experience.

The evaluation also highlighted opportunities to improve user engagement and aesthetic consistency through features like gamification and personalization, which could significantly enhance the app’s adoption and impact.

A significant limitation of this study was the lack of patient involvement in the evaluation phase. This decision prioritized input from health care professionals to ensure alignment with clinical and educational standards. Ongoing clinical trials involving patients with cancer are addressing this limitation by capturing patient-specific feedback on usability and impact using the uMARS tool.

This study contributes to the growing field of mHealth by underscoring the importance of phased evaluations that integrate iterative testing and stakeholder feedback. Future work will focus on refining the app based on patient insights, ensuring it effectively meets its target population’s needs and improves the oncology care experience.

### Limitations and Future Directions

The primary limitation of this study was the exclusion of patients with cancer during the initial evaluation phase. This decision was based on the need to ensure the app met clinical and educational standards before involving end-users. Health care professionals were selected as evaluators due to their expertise in oncology care and their ability to provide detailed feedback on the app’s content and usability.

Efforts were made to recruit patients for this phase through oncology clinics and patient support groups. However, challenges such as scheduling conflicts, the burden of ongoing treatments, and limited interest in participating in a non-clinical evaluation restricted patient involvement. Additionally, it was not possible to recruit patients who were not already participating in other clinical trials, as this could have introduced confounding variables to this study.

To address this limitation, 2 clinical trials are currently underway, incorporating the uMARS tool to assess the app’s usability and quality from the patient perspective. These trials will provide a comprehensive understanding of the app’s impact and inform future refinements.

By prioritizing health care professionals in the early phases, we ensured a solid foundation for the application’s content and usability, which will now be validated and refined based on direct input from patients.

## Abbreviations

CLS: cumulative layout shift
DALYs: disability-adjusted life years
DDoS: distributed denial-of-service
FCP: first contentful paint
ICC: interclass correlation coefficient
LCP: large contentful paint
MARS: Mobile App Rating Scale
PREMs: Patient-Reported Experience Measures
PROMs: Patient-Reported Outcome Measures
SEO: search engine optimization
TBT: total blocking time
WHO: World Health Organization
